# Perspectives of patients with colorectal cancer liver metastases on e-consultation in transmural care: a qualitative study: Is privacy really an issue?

**DOI:** 10.1186/s12913-023-09408-5

**Published:** 2023-05-25

**Authors:** T. Hellingman, M. L.H. van Beneden, C. M. den Bakker, B. M. Zonderhuis, G. Kazemier

**Affiliations:** 1 Department of Surgery, Cancer Center Amsterdam, Amsterdam UMC, Vrije Universiteit Amsterdam, de Boelelaan 1117, 1081 HV Amsterdam, The Netherlands; 2 Department of Strategy and Innovation, Cancer Center Amsterdam, Amsterdam UMC, Vrije Universiteit Amsterdam, de Boelelaan 1117, 1081 HV Amsterdam, The Netherlands; 3 Department of Surgery, Spaarne Gasthuis, Boerhaavelaan 22, 2035 RC Haarlem, The Netherlands

**Keywords:** eHealth, Telecare, privacy, Patients’ views

## Abstract

**Background:**

Comprehensive cancer networks have been established to deliver high-quality care for patients with cancer. Logistic challenges are faced, when patients need to be referred for specialized treatments. Despite strengthened privacy legislations, digital platforms are increasingly used to consult specialists from dedicated liver centers or refer patients with colorectal cancer liver metastases (CRLM) for local treatment strategies. This qualitative study aimed to explore the perspectives of patients with CRLM regarding e-consultation of transmural specialists.

**Methods:**

A focus group study was conducted. Patients referred from regional hospitals to an academic liver center for treatment of CRLM were asked to participate. Focus group discussions were audio-recorded and transcribed verbatim. A thematic content analysis of data was conducted, comprising open, axial, and selective coding of the transcripts. The consolidated criteria for reporting qualitative research (COREQ) were used.

**Results:**

Two focus groups were held, involving 11 patients and 8 relatives. Three major themes were identified with regard to e-consultation in transmural care: ‘data management’, ‘expertise’, and ‘information and coordination’. Confidence in the expertise of physicians appeared most important during the course of treatment, as patients experienced uncertainty after diagnosis of cancer. Despite the privacy risks, use of digital communication platforms to contact experts in the field were strongly endorsed to improve eligibility for potentially curative treatment. Moreover, e-consultation of specialists may reduce waiting times, due to effective coordination of care.

**Conclusion:**

Initiatives to improve medical data transfer between care providers were encouraged to achieve effective coordination of oncological care. The potential hazard of privacy violation associated with digital data exchange is accepted by patients and their relatives, provided that use of digital data improves patient’s own health care, research or education.

**Supplementary Information:**

The online version contains supplementary material available at 10.1186/s12913-023-09408-5.

## Background

Diagnosis and treatment of cancer is complex and often requires involvement of multiple specialists of various disciplines. Exponential increase in medical knowledge and technical advances resulted in further specialization of oncological care. Moreover, centralization of high-complex procedures improved outcome of patients suffering from cancer [1–4]. Consequently, consultation between clinicians became more important, even between different hospitals boosting transmural oncological care (i.e. care beyond the walls of a hospital). Individual hospitals more frequently lack cancer specific expertise and need to refer patients for specialized cancer treatments. Logistic difficulties in health data transfer may result in time-consuming procedures and hamper decision-making, while prolonged time to diagnosis and treatment is related to worse prognosis [5, 6]. Coordination and effective communication between hospitals is therefore essential to prevent hospital delay and improve outcome of patients with cancer [7].

In the current digital era, several eHealth solutions have been implemented to improve oncological care by managing health data transfer. Initiatives, like electronic patients files, e-consultation of specialists, and use of digital platforms to share health data have been introduced [8–14]. However, worldwide increased privacy concerns resulted in more restricting privacy legislations [15, 16]. In April 2016, the European Union passed the General Data Protection Regulation to protect natural persons with regard to the processing of personal data and on the free movement of such data [17]. The tension between preserving the privacy and confidentiality of patients and the need to access medical data to deliver high-quality care is ever since growing [18–20].

Patients with colorectal cancer liver metastases (CRLM) are commonly diagnosed in (regional) hospitals by specialists with expertise in colorectal cancer, while local treatment (i.e. resection and/or ablation) of CRLM is performed in dedicated liver centers by liver surgeons and/or interventional radiologists specialized in ablation of liver tumors [21, 22]. Patients are referred to those dedicated liver centers when local treatment of CRLM is deemed feasible. When local treatment is not deemed feasible, patients may receive (palliative) systemic therapy from medical oncologists in (regional) hospitals specialized in colorectal cancer nearby home. To select patients eligible for referral, treatment strategy is often assessed during e-consultation, either by video-conferencing during multidisciplinary team meetings or assessment by an online expert panel through a digital communication platform [14]. Several challenges are faced under current legislation, as specific written permission is required to share health data with specialists from other hospitals [17, 23]. In addition, a personalized treatment plan is often obtained by multiple experts, while no patient-doctor relationship has been established yet. In this context, relevant questions with regard to e-consultation of specialists from other hospitals arise, like who should report back to whom? How to deal with non-consensus? And is documentation allowed? The purpose of this study was to explore the perspectives of patients with CRLM regarding e-consultation of transmural specialists, including the associated privacy risks.

## Methods

This study was conducted as part of the Moving Towards Regional Oncology Networks program, a nationwide initiative of the Dutch Ministry of Health, Welfare and Sport to optimize oncological care. Among others, digital data transfer and e-consultation of online expert panels were introduced by the program to achieve equal, high-quality and more efficient oncological care in community and academic hospitals.

### Design

A qualitative focus group study was conducted to explore perspectives of patients with regard to e-consultation in transmural care. This design encourages interaction between participants by enabling them to exchange anecdotes and to respond to each other’s experiences and point-of-views [24]. Hereby, data could be obtained on how and why people think a certain way, e.g. with regard to privacy risks associated with e-consultation. In addition, a qualitative description of the findings derived from thematic analysis was conducted, as the primary goal of this study was to explore participants’ views rather than quantify content of data [25]. The guidelines of the consolidated criteria for reporting qualitative research (COREQ) were used (See Additional file 1) [26].

### Participant selection and recruitment

A purposive selection of patients diagnosed with CRLM in a non-liver center and referred for treatment of CRLM to VU University Medical Center (an academic liver center) was conducted. Patients were identified from electronic patient files after visiting the out-patient clinic between January 2016–2017. Patients were contacted by phone and asked to participate by a member of the healthcare team (BZ and GK) or a medical researcher (TH) after approval by the head practitioner. When patients wanted to participate, a confirmation with detailed information about the content of the focus group was sent by mail. Patients were asked to bring a relative to the focus group meetings for support. Participants were not selected on the basis of sex, race, religious belief, age, nor known experiences or satisfaction with regard to diagnosis and treatment in transmural care.

### Setting

Focus groups were held in the academic referral liver center in Amsterdam, the Netherlands. Participants received a brief overview on the use of e-consultation in current clinical practice, including an introduction to the concept of online expert panels, by one of the liver surgeons (BZ) prior to the focus group discussions. A female quality improvement manager specialized in patient and family participation, with many years of experience and trained in qualitative research, moderated the two focus group discussions (MB). No relationship between the moderator and the participants, nor a specific interest in this topic was established prior to the study commencement. A female researcher and project employee, who implemented an online expert panel for patients suffering from CRLM, attended the focus group discussions as listener (TH), and was positioned outside the group.

### Data collection

An interview guide, mainly consisting of open-ended questions based on previous research and current privacy regulations, was drafted by the project team prior to the focus group meetings (See Additional file 2) [23]. These questions were ordered by theme and served to facilitate discussions, instead of simply interviewing the participants. Focus groups of 45–75 min were audio-recorded and transcribed verbatim. Written consent to record sessions were obtained prior to the start of focus group discussions. No field notes, besides the positioning of participants, were made. No repeat interviews were carried out. Returning of transcripts was performed on demand. Research was performed in accordance with the Declaration of Helsinki. The protocol was approved by the Ethical Review Board of Amsterdam UMC, location VUmc (registration number 2020.010) and written informed consent to publish findings was obtained from all participants.

### Data analysis

Transcripts were read and reread by two researchers (TH and MB), both certified in qualitative research, to familiarize with the data. Data were analyzed in three phases by open, axial, and selective coding in Atlas.ti (version 8) followed by discussion. A thematic map was generated, identifying themes and subthemes after open and axial coding. Potential themes from the predefined topic list were reviewed and new themes were identified by an inductive approach. Based on the thematic map, a coding tree was generated, and transcripts were recoded selectively (see Additional file 3). Data saturation (i.e. code saturation) on each theme was discussed between researchers after the second focus group meeting. No feedback on the findings was provided by participants.

## Results

### Participant characteristics

Twenty-seven patients were invited to participate in this study. In total, two focus groups were held, involving 11 patients and 8 relatives (see Table 1). Median age of participants was 64 years [range: 28–80]. Patients were referred to the academic liver center from four regional hospitals. The majority of patients were male and not treated with systemic therapy for CRLM. About half of these patients experienced recurrent disease at the time of conducting this study.

**Table 1:** Characteristics of participants

		Sex	Age	Referral hospital	Months from liver surgery	Systemic treatment	Setting	Recurrence
**Focusgroup 1**								
Participant #1	Patient	m	59	#1	9	no		yes
Participant #2	Patient	f	64	#2	11	no		no
Participant #3	Relative	m	63					
Participant #4	Relative	f	70					
Participant #5	Patient	m	73	#3	10	no		yes
Participant #6	Patient	f	74	#4	3	no		no
Participant #7	Patient	m	72	#3	9	no		no
Participant #8	Relative	f	57					
**Focusgroep 2**								
Participant #9	Relative	m	28					
Participant #10	Patient	m	57	#4	36	no		no
Participant #11	Patient	m	59	#4	12	yes	palliative	yes
Participant #12	Patient	m	80	#3	6	no		no
Participant #13	Relative	f	80					
Participant #14	Patient	m	77	#4	2	no		no
Participant #15	Relative	f	71					
Participant #16	Patient	f	60	#3	6	yes	neoadjuvant + palliative	yes
Participant #17	Relative	f	62					
Participant #18	Relative	m	38					
Participant #19	Patient	m	75	#3	15	yes	neoadjuvant	yes

### E-consultation in transmural care

Three main themes were identified from the focus group discussions with regard to e-consultation in transmural care: (1) data management, (2) expertise, and (3) information and coordination. Data saturation was reached after two focus group meetings, as no new topics (i.e. (sub)themes) were introduced and sufficient data was collected. Findings are summarized in Table 2.

**Table 2:** Summary of results on e-consultation in transmural care

	Pros	Cons and needs
Data management	Advances in own health, education or research	Privacy hazard
Expertise	More easily accessible Contact multiple specialists in the field Eligibility for potentially curative treatment Trust in assessment of treatment strategy	Challenges in case of non-consensus Need for information about expertise of specialists
Information and coordination	Potentially reduce waiting times No specific approval deemed necessary	Keep patients informed Ambiguity about head practitioner Communication on patient level

### Data management

Digital data exchange was deemed inevitable in the current digital era. Regardless, a positive attitude towards this development was expressed. Digital data exchange was believed to make data management quicker and easier, especially in case of transmural care. Associated privacy aspects were discussed and some requirements for the use of digital communication platforms were set to avoid potential data breach:

- digital communication platforms should be properly secured.
- medical data should only be shared among doctors or healthcare professionals.

In particular, unwanted data revelation to healthcare and life insurance agencies or potential employers was a topic of concern. However, some patients did not worry about the hazard of privacy violation, as sharing medical data is obligatory while taking out (life) insurances. So, a potential data breach should have no consequences.

“ Yes, but the insurance company sends me a form and on that form I have to write down whether I have an illness or not. So if I don’t, I’m breaking the law.”RS#10.

Even though no consensus about the impact of a potential data breach was obtained, the hazard was clearly subservient to the process of ‘getting better’. Participants mentioned that the goal of privacy was to protect the patient, while strengthened privacy regulations may interfere with efficient coordination of medical care. When in bad health, recovery appeared the primary objective rather than worrying about privacy concerns.

“I find privacy a difficult topic to discuss, as privacy is intended to protect the individual, but health care is also for the individual. One thing is very important to me, the exchange of medical data can make or break success. So, I believe exchange of data is crucial and more important than a potential privacy breach.”RS#1.

As data exchange appeared crucial in transmural care, a potential hack was considered less important than sharing medical data to receive better care. A central database with a national patient record was even suggested to improve secured communication between hospitals.

“I think you need to prefer a single patient file, which you can take with you, wherever you are treated.”RS#11.

Medical data sharing and documentation needed to serve a purpose. Patients’ files could be used for patient’s own health, education or research. The use of digital platforms to transfer medical data was supported to enhance oncological care and improve lead time for patients with CRLM. Other e-Health initiatives were encouraged, as long as their objective was to improve cancer treatment or future perspectives.

“From my perspective, all kinds of information is allowed that ensures me and all those other patients who get cancer, if it facilitates better and quicker treatment or a better way to get your health back.”RS#10.

### Expertise

Participants primarily focused on recovery after cancer diagnosis. The main priority was to become eligible for potentially curative treatment strategies, in order to regain their health and improve overall survival.

“My primary interest is to determine the most optimal treatment strategy based on my data, which may increase my chance on survival.”RS#1.

“Life expectancy is ofcourse very important to us.”RS#14.

Consequently, expertise of liver specialists appeared important in treatment decision-making. Consultation of multiple liver specialists and a weighted advice on treatment strategy was highly appreciated and recommended by the participants.

“Well, we already said we were positive about the fact that more than one or two people assess treatment strategy and multiple professionals discuss your particular situation.”RS#14.

E-consultation of these experts was encouraged, as deliberation with multiple liver specialists improved trust and assurance in treatment of CRLM. Live discussion between specialists during multidisciplinary team meetings by video-conferencing was considered favorable, as compared to a non-simultaneous approach. The status and quality of consulted specialists were of interest, rather than hospital of employment or previous established patient-physician relationship.

“Most of the time there is nothing to see on the outside. They may look into your beautiful eyes, but that doesn’t help either.”RS#4.

Whilst expertise of liver specialists was considered important, patients were particularly interested in finding a surgeon who deemed local treatment feasible, as local treatment of CRLM is the only potentially curative treatment. Participants believed that use of e-consultation services might bring eligibility for local treatment strategies closer. Expansion of an expert panel in case of non-consensus was suggested, especially when the majority of panelists advised against upfront local treatment of CRLM. Participants even suggested to look for expertise abroad, if they were deemed ineligible for local treatment of CRLM.

“I see the expert panel as a second opinion.”RS#12.

E-consultation of liver specialists was seen as a second opinion. High expectations of academic liver specialists were discussed, as they were believed to have more expertise, based on employment in high-volume referral centers with attention for education. Consultation of these specialists was considered valuable during assessment of treatment strategy.

“If they say: well, we will discuss your case with a few people, is very different from when they say: well, we have the panel, an expert panel and they’re going to look at your case again. That gives me more hope.”RS#19.

### Information and coordination

Effective coordination and communication with the patient and between specialists was considered pivotal to deliver high-quality care. Information about diagnosis and treatment after e-consultion of experts should preferably be given by the head practitioner. According to the focusgroup discussions, reports should not be send to the patient directly, as the patient would not be able to understand ‘the language’ and nuance of specialists’ recommendations. For both patients and their relatives, it was not always clear who the head practioner was during the course of treatment in transmural care. So, some requirements were set.

The head practioner should:

- be acquinted with the patient.
- be familiar with the medical data.
- be able to deliberate on choice of treatment.

“…the treatment plan should be discussed with the person who can explain it to me, with whom I can engage in a discussion and with whom I can make a decision.”RS#1.

However, details about the advised treatment, including complication risk and potential side effects, should be given by the attending physician. For example: the surgeon who will perform the surgery or the medical oncologist who will administer chemotherapy. Communication should be open, honest and on patient level, so professional jargon should be avoided and speech speed should be adjusted.

“Once, we have had a doctor, who spoke very quickly and often threw in medical terms, so I asked: can’t you speak slower?”RS#4.

“It is complicated, because there are so many involved people, with some you get along better, get information at your own level of thinking, while others are more clinical and distant.”RS#6.

The general practioner (GP) was not deemed suitable, as most questions after e-consultation appeared treatment-specific or related to the logistics of outpatient visits. Timely communication between the hospital and the general practitioner often appeared a challenge, while participants expected the GP to be informed at all times.

“I would prefer to receive information from the surgeon, who is going to perform the surgery, but I think the GP should be informed as well, about what is going to happen, since she needs to be able to act on the situation at all times.”RS#2.

“My GP is well informed, but often too late.”RS#5.

Patients with CRLM underwent a strict follow-up schedule after local treatment of CRLM. Diagnostic imaging and blood samples were taken every three months for the first year. During this period, participants lived between hope and fear as many patients suffer from recurrent disease. The average waiting time between examinations and official results was one to two weeks in current clinical practice. Reduced waiting time was preferred, due to uncertainty about the patient’s health. A waiting period of two to seven days was considered acceptable. Participants encouraged the use of digital communication platforms to achieve this goal. Although some patients preferred an even shorter waiting period, consensus about lead time was eventually reached as speed could interfere with quality of care.

“… the shorter the waiting time the better.”RS#1.

“Yes, but the shorter the better is not always the case. You should have a more certain result rather than a very quick result that probably deviates from the truth. So I think, and this research or this panel is very good for that, doctors in other hospitals may have access to data sooner and can reach a conclusion more quickly.”RS#3.

No consensus was reached about feedback on a non-unanimous decision. Participants from the first focus group wanted to receive all information to make an informed decision on treatment strategy, while some participants from the second focus group expected an unambiguous advise.

“I think it also differs from patient to patient, because some patients say: I don’t want to know. They’re just scared or something… I think it’s a little, yes that might sound a little weird, that the doctor should know how the patient wants to be informed.”RS#8.

In case of non-consensus, this information should be kept as it may cause confusion and commotion. However, when (palliative) systemic therapy was advised after e-consultation of specialists, participants did want to know if consensus was reached, in order to pursue treatment by those who deemed local treatment feasible.

“… oh yes, sometimes you are brought into doubt, and think o help, two say this, while two others say that, what now?”RS#2.

“It creates anxiety, yes, because if one says: it is easy to perform the surgery, and the other says: well, there are still some complications… You’re a layman aren’t you, you’re the one with cancer, of which you know nothing about. And those people are, as you also say, people who are trained, who have experience. They can judge. But if they do not agree 100% as a group, then confusion ensues.”RS#15.

Patients wanted to be informed about consultation between clinicians, whether this consultation consisted of a face-to-face meeting within the hospital or an e-consultation with a (virtual) tumor board. Moreover, additional information about the expertise of these consultants was desired to increase confidentiality in the course of treatment. A short overview on their training and achievements in an information leaflet or on the hospital’s website was proposed. No specific written approval for (e-)consultation or sharing medical data was deemed necessary, as participants assumed doctors would act in their best interest.

“The profession of doctors is that they should make you better, they should cure you. So, I trust the advice of a doctor, since I assume it will be the best treatment for me. For me personally, I don’t want to know anything more, besides the expected time and place.”RS#11.

## Discussion

This study showed a positive attitude towards e-consultation services in transmural care among patients suffering from CRLM, as expertise from various specialists was assumed to become more easily accessible. Consequently, eligibility for potentially curative treatment was considered more feasible. The potential hazard of privacy violation associated with digital data exchange was accepted, as long as the use of digital data supported a higher purpose, like improving patient’s health, education or research. Digital data transfer could potentially improve patient care by reducing waiting times. Hereby, uncertainty during the course of treatment and follow-up procedures could be reduced.

Implementation of eHealth initiatives, like e-consultation and digital data exchange was supported by patients and their relatives. Patients wanted to be informed about the use of these services, however specific written approval was not deemed necessary as this might interfere with the workflow. Surprisingly, a nationwide electronic health record was suggested, while implementation of a nationwide personal health record was previously disapproved by the Dutch Government, mainly based on the hazard of privacy violation [27].

E-consultation in ambulatory and transmural care for patients in need of specialized expertise has been widely adopted. Perspectives and satisfaction of providers (i.e. GPs and clinicians) have been researched extensively, while studies on the perspectives of patients are scarce [28–35]. Most studies focused on e-consultation models in ambulatory care, as an alternative to face-to-face visits. Based on these qualitative studies, faster and improved access to specialist care were acknowledged as favorable outcomes [35–38]. However, to the best of our knowledge, no study assessed patient’s views on e-consultation between secondary and tertiary care yet.

Ackerman et al. studied patient’s perspectives on e-consultation in an academic setting. Strong support for e-consultation between the primary care physicians and academic specialists was expressed, in particular due to the benefit of more rapid access to specialist expertise. In contrast to the present findings, no consensus about the extent to which patients should be involved in the decision to use e-consultation was reached. Moreover, even though trust and reliance in physicians came forward during the focus group discussions, Ackerman et al. observed disagreement about the language to explain specialist’s recommendations. About half of the participants wanted to access specialist’s verbatim response, while others preferred the primary care clinician’s summary in order to understand it better [36]. Joschko et al., showed that 93% of participants expressed no concerns about potential privacy issues as their information was being shared electronically. Moreover, in accordance with findings of the current study, participants expressed feelings of reassurance after e-consultation of specialists, since more than one person was involved in making the decision [37]. Based on the findings of this study, strengthened privacy regulations in e-consultation and digital exchange of medical data should be loosened to benefit patients in transmural care. Complex discussions on liability and who is ultimately delivering care should make room for what is best for the patient. However, with regard to these questions, current practice asks for definitive regulatory guidelines [23, 38]. Moreover, health systems demand requirements to ensure quality of care and prevent potential misuse.

Several measures were taken to improve qualitative rigour and trustworthiness of this study, including deductive and inductive coding by multiple coders, and a thick description of the data to promote reliability, credibility and validity [39]. Physicians were not allowed during the focus group discussion as participants may feel uncomfortable expressing negative emotions. However, one of the project employees (TH), positioned outside the focus group, attended the focus group discussions to familiarize with the data (i.e. their points-of-view and motivation). Patients were aware of her presence, which may have hampered honest conversation. Moreover, generalizability of findings is a concern based on the design of the study. Findings may be subject to selection bias, as all patients were considered for curative treatment strategies, so ultimately benefited from e-consultation of liver surgeons in a tertiary referral hospital. In addition, patients were asked to bring a relative for support and to participate in these focus group meetings, which resulted in a less homogenous study group. Invitation of these relatives, who may represent the healthier population, could have contributed to generalizability of findings. However, the majority of questions were patient specific, so input came primarily from patients, resulting in findings reflecting primarily patients’ views. This may also explain why data saturation was reached after two relatively short focus group discussions. No analysis on demographic strata was carried out. Selection of a study population solely consisting of patients, more often treated in a palliative setting, or with less life threatening conditions, may have benefited the study. Last, focus group discussions were conducted in Dutch. Translation may have resulted in some loss of content.

Based on the findings of the current study, future research should focus on successful implementation of eHealth initiatives to further optimize transmural care, whilst respecting (inter)national rules and regulations to protect personal data. Clinical practice is currently centered around the patient, as opposed to a system orientated approach [38]. Several use-cases to control medical data transfer have been introduced in Europe, like personal and Cloud-based selective authentic electronic health records [8, 40]. Although implementation of a personal electronic health record was supported by participants of the current study, particularly to ensure up-to-date information on patient’s health, interoperability remains a topic of concern. Whether current findings relate to other (patient) populations should be further explored. In addition, positive effects on quality of life and psychological effects due to improved waiting times need to be clarified [6].

With the current Covid-19 pandemic, e-consultation services and digital data transfer have been increasingly used to deliver care, as face-to-face visits are limited and patients are advised against traveling. E-consultation of specialists may hereby ensure timely access to specialized care. Current findings support the use of these services also for cancer patients outside the current Covid-19 pandemic. However, even though patients want to be informed, specific approval to use these services is not deemed necessary. To further improve transmural care, in particular waiting times, current rules and regulations with regard to privacy aspects should therefore be loosened.

## Conclusions

E-consultation between specialists in transmural care is considered an acceptable and effective approach to deliver oncological care for patients suffering from CRLM, in particular to access specialized liver expertise and potentially reduce waiting times. The potential hazard of privacy violation was accepted, considering digital platforms met the set requirements. Moreover, the added value of these services outweighed the negatives. In particular, advances in patient’s own health care, research and education were mentioned.

## Electronic supplementary material

Below is the link to the electronic supplementary material.

Supplementary Material 1

Supplementary Material 2

Supplementary Material 3

## Data Availability

The data that support the findings of this study are available from the corresponding author (TH; t.hellingman@amsterdamumc.nl), but restrictions apply to the availability of these data, which were used under license for the current study, and so are not publicly available. Data are however available from the authors upon reasonable request and with permission of the participants.

## References

[1] Sheetz KH, Dimick JB, Nathan H. Centralization of High-Risk Cancer Surgery Within Existing Hospital Systems. J Clin oncology: official J Am Soc Clin Oncol. 2019:Jco1802035. 10.1200/JCO.18.02035 PMC7351344 31251691

[2] Fischer C, Lingsma H, Klazinga N, Hardwick R, Cromwell D, Steyerberg E (2017). Volume-outcome revisited: the effect of hospital and surgeon volumes on multiple outcome measures in oesophago-gastric cancer surgery. PLoS ONE.

[3] Vos M, Blaauwgeers HGT, Ho VKY, van Houdt WJ, van der Hage JA, Been LB (2019). Increased survival of non low-grade and deep-seated soft tissue sarcoma after surgical management in high-volume hospitals: a nationwide study from the Netherlands. Eur J cancer (Oxford England: 1990).

[4] Bruins HM, Veskimae E, Hernandez V, Neuzillet Y, Cathomas R, Comperat EM et al. The Importance of Hospital and Surgeon Volume as Major Determinants of Morbidity and Mortality After Radical Cystectomy for Bladder Cancer: A Systematic Review and Recommendations by the European Association of Urology Muscle-invasive and Metastatic Bladder Cancer Guideline Panel. European urology oncology. 2019. 10.1016/j.euo.2019.11.005 31866215

[5] Khorana AA, Tullio K, Elson P, Pennell NA, Grobmyer SR, Kalady MF (2019). Time to initial cancer treatment in the United States and association with survival over time: an observational study. PLoS ONE.

[6] Neal RD, Tharmanathan P, France B, Din NU, Cotton S, Fallon-Ferguson J (2015). Is increased time to diagnosis and treatment in symptomatic cancer associated with poorer outcomes? Systematic review. Br J Cancer.

[7] Walsh J, Young JM, Harrison JD, Butow PN, Solomon MJ, Masya L (2011). What is important in cancer care coordination? A qualitative investigation. Eur J Cancer Care.

[8] Poss-Doering R, Kunz A, Pohlmann S, Hofmann H, Kiel M, Winkler EC (2018). Utilizing a prototype patient-controlled Electronic Health Record in Germany: qualitative analysis of user-reported perceptions and perspectives. JMIR formative research.

[9] Pitter JG, Csanadi M, Szigeti A, Lukacs G, Kovacs A, Moizs M (2019). Planning, implementation and operation of a personalized patient management system for subjects with first suspect of cancer (OnkoNetwork): system description based on a qualitative study. BMC Health Serv Res.

[10] Funderskov KF, Boe Danbjorg D, Jess M, Munk L, Olsen Zwisler AD, Dieperink KB (2019). Telemedicine in specialised palliative care: Healthcare professionals’ and their perspectives on video consultations-A qualitative study. J Clin Nurs.

[11] Duineveld LA, Wieldraaijer T, Wind J, Verdonck-de Leeuw IM, van Weert HC, van Uden-Kraan CF (2016). Primary care-led survivorship care for patients with colon cancer and the use of eHealth: a qualitative study on perspectives of general practitioners. BMJ open.

[12] Vimalananda VG, Gupte G, Seraj SM, Orlander J, Berlowitz D, Fincke BG (2015). Electronic consultations (e-consults) to improve access to specialty care: a systematic review and narrative synthesis. J Telemed Telecare.

[13] Sunderland M, Teague R, Gale K, Rademaker M, Oakley A, Martin RCW. E-referrals and teledermatoscopy grading for melanoma: a successful model of care. Australas J Dermatol. 2020. 10.1111/ajd.13230 32064590

[14] Hellingman T, Swart ME, Meijerink MR, Schreurs WH, Zonderhuis BM, Kazemier G. Optimization of transmural care by implementation of an online expert panel to assess treatment strategy in patients suffering from colorectal cancer liver metastases: A prospective analysis. J Telemed Telecare. 2020:1357633x20957136. 10.1177/1357633X20957136 33019855

[15] Holmes JH, Soualmia LF, Seroussi B (2018). A 21st century embarrassment of riches: the balance between Health Data Access, usage, and sharing. Yearb Med Inform.

[16] Olver I (2011). Improving cancer treatment by addressing legislative and regulatory issues. Public Health.

[17] EUR-lex. General Data Protection Regulation 2016 [cited 2016. Available from: https://eur-lex.europa.eu/eli/reg/2016/679/2016-05-04.

[18] Mold F, Hendy J, Lai YL, de Lusignan S (2019). Electronic Consultation in Primary Care between Providers and Patients: systematic review. JMIR Med Inf.

[19] Osman MA, Schick-Makaroff K, Thompson S, Bialy L, Featherstone R, Kurzawa J (2019). Barriers and facilitators for implementation of electronic consultations (eConsult) to enhance access to specialist care: a scoping review. BMJ global health.

[20] Qi M, Cui J, Li X, Han Y (2021). Perceived factors influencing the Public Intention to Use E-Consultation: analysis of web-based Survey Data. J Med Internet Res.

[21] Young AL, Adair R, Culverwell A, Guthrie JA, Botterill ID, Toogood GJ (2013). Variation in referral practice for patients with colorectal cancer liver metastases. Br J Surg.

[22] Lam-Boer J, van der Stok EP, Huiskens J, Verhoeven RH, Punt CJ, Elferink MA (2017). Regional and inter-hospital differences in the utilisation of liver surgery for patients with synchronous colorectal liver metastases in the Netherlands. Eur J cancer (Oxford England: 1990).

[23] Hellingman T, Tiems SF, Brakel LF, Kazemier G. [Sharing oncological knowledge via expert panels; are there legal obstacles or practical objections?]. Ned Tijdschr Geneeskd. 2019;163. 31050265

[24] Kitzinger J (1995). Qualitative research. Introducing focus groups. BMJ (Clinical research ed).

[25] Vaismoradi M, Turunen H, Bondas T (2013). Content analysis and thematic analysis: implications for conducting a qualitative descriptive study. Nurs Health Sci.

[26] Tong A, Sainsbury P, Craig J (2007). Consolidated criteria for reporting qualitative research (COREQ): a 32-item checklist for interviews and focus groups. Int J Qual health care: J Int Soc Qual Health Care.

[27] Staten-Generaal EKd. Eerste Kamer verwerpt unaniem voorstel landelijk EPD. 2011.

[28] Anderson D, Porto A, Koppel J, Macri G, Wright M (2020). Impact of Endocrinology eConsults on Access to Endocrinology Care for Medicaid Patients. Telemedicine J e-health: official J Am Telemedicine Association.

[29] Keely E, Williams R, Epstein G, Afkham A, Liddy C (2019). Specialist perspectives on Ontario Provincial Electronic Consultation Services. Telemedicine J e-health: official J Am Telemedicine Association.

[30] Lee M, Leonard C, Greene P, Kenney R, Whittington MD, Kirsh S (2020). Perspectives of VA Primary Care Clinicians toward Electronic Consultation-Related workload burden: a qualitative analysis. JAMA Netw open.

[31] Liddy C, Afkham A, Drosinis P, Joschko J, Keely E (2015). Impact of and satisfaction with a New eConsult Service: a mixed methods study of primary care providers. J Am Board Family Medicine: JABFM.

[32] Liddy C, Sethuram C, Mihan A, Moroz I, Keely E. Primary Care Providers’ Perspectives on the Ontario eConsult Program. Telemedicine J e-health: official J Am Telemedicine Association. 2020. 10.1089/tmj.2020.0338 33252320

[33] Zallman L, Fisher CF, Ladner S, Mengistu K, Rapaport AB, Bor D (2020). Inter-clinician eConsults without programmatic incentives or requirements: a qualitative study of primary care provider perspectives. Fam Pract.

[34] Atherton H, Brant H, Ziebland S, Bikker A, Campbell J, Gibson A et al. Health Services and Delivery Research. The potential of alternatives to face-to-face consultation in general practice, and the impact on different patient groups: a mixed-methods case study. Southampton (UK):NIHR Journals Library. 29889485

[35] Bello AK, Molzahn AE, Girard LP, Osman MA, Okpechi IG, Glassford J (2017). Patient and provider perspectives on the design and implementation of an electronic consultation system for kidney care delivery in Canada: a focus group study. BMJ open.

[36] Ackerman SL, Dowdell K, Clebak KT, Quinn M, Shipman SA (2020). Patients assess an eConsult Model’s acceptability at 5 US Academic Medical Centers. Ann Fam Med.

[37] Joschko J, Liddy C, Moroz I, Reiche M, Crowe L, Afkham A (2018). Just a click away: exploring patients’ perspectives on receiving care through the Champlain BASETM eConsult service. Fam Pract.

[38] Liddy C, Hauteclocque J, Moroz I, Oppenheimer L, Sturge D, Burns KK et al. Enabling patient-centred policy for electronic consultations: A qualitative analysis of discussions from a stakeholder meeting. J Telemed Telecare. 2020:1357633x20926779. 10.1177/1357633X20926779 32486888

[39] Rendle KA, Abramson CM, Garrett SB, Halley MC, Dohan D (2019). Beyond exploratory: a tailored framework for designing and assessing qualitative health research. BMJ open.

[40] Alaqra AS, Fischer-Hübner S, Framner E (2018). Enhancing privacy controls for patients via a selective authentic Electronic Health Record Exchange Service: qualitative study of perspectives by Medical Professionals and Patients. J Med Internet Res.

