# Supplementary material for: Perspectives of patients with colorectal cancer liver metastases on e-consultation in transmural care: a qualitative study: Is privacy really an issue?
Source: BMC Health Serv Res. 2023 May 25;23:541. doi: 10.1186/s12913-023-09408-5 (PMC10209560; doi:10.1186/s12913-023-09408-5)
Supplement: Supplementary file 2 — Supplementary Material 2 [file 12913_2023_9408_MOESM2_ESM.pdf]

Focus group 'developments in oncological care and patient privacy'
VU university medical center, Amsterdam

Motive
Development of online expert panels to optimize transmural care by:
- reducing potential under- and overtreatment of patients with cancer
- sharing medical data without pre-established patient-physician relationship

Purpose
To explore the points-of-view of patients and their relatives on sharing confidential data through digital communication platforms with specialists without patient-physician relationship under current legislation

1. Logistics from diagnosis to treatment <u>Discussed in:</u>

Sample questions:

1. How did you experience the process of diagnosis and treatment? FG1/FG2
2. How did you experience waiting times? And why? FG1/FG2
3. What seems an appropriate waiting time for you? And why? FG1/FG2
4. How was (further) treatment in (or consultation of) a different hospital communicated with you? How did you feel about that? FG1/FG2
5. Is receiving care in the 'right' place always important? Or are there objections? FG1/FG2
6. If there are objections, what are they? -
   Example: travelling distance? What is the maximum acceptable distance from home to receive care? And why is that? Traveling distance for you or for your relatives?

Information needed:
Views on experienced and acceptable waiting times FG1/FG2
Views of patients and their relatives on: the right care in the right place FG1/FG2

2. Privacy
Sharing medical data (confidential)

Pros:
- No compact disks delivered by courier (less susceptible to unauthorized reading)

Cons:
- Imaging in the cloud, risk for data breach
- Imaging stored on server for 3 months

Sample questions:

1. How do you feel about privacy? FG1/FG2
2. How do you feel about sharing medical information with others through the cloud? FG1/FG2
3. Are their requirements? FG1/FG2
4. Do you agree with current restricting regulations? Why? FG2

Information needed:
Views of patients and relatives on sharing (medical) data among clinicians FG1/FG2
Requirements to share medical data FG1/FG2
The role of the provider in their considerations -

3. Legal aspects

Assessment of treatment strategy without pre-estbalished patient-doctor relationship
Able to make an informed decision, due to access to (factual) medical information
Issue for both video-conferencing and online expert panels

Sample questions:

1. How do you feel about receiving treatment advice from an unacquainted specialist? Are there objections? FG1/FG2
2. If there are objections, are there requirements that can be set to advice on treatment strategy? FG1/FG2
3. The duty to file is in conflict with the identification requirement, may data be filed or should it be destroyed? Why? FG1/FG2
4. If data may be filed, which data and time period is allowed? FG1/FG2

Information needed:

Concerns about treatment advice from an expert from another hospital	FG1/FG2
Views on filing/storing data	FG1/FG2

4. Involvement

Sample questions:

1. How did you experience shared decision-making? Were all treatment options discussed with you? Do you wish to be more involved in decision-making on treatment strategy? FG2
2. If there is no consensus regarding your treatment advice, would you like to know? Why? FG1/FG2
3. How do you feel about contact with the general practitioner (GP)? Is he/she involved? FG1/FG2
4. Are you satisfied with the participation of the GP during the process of diagnosis and treatment? FG1/FG2
5. If you desire more involvement, what would be ideal for you? And are you able to elaborate on why this is not the case? Is this the responsibility of de GP, the medical specialist or yourself? FG1/FG2

Information needed:

Views and experiences on shared-decision making	FG2
Communication regarding treatment advice	FG1/FG2
Views and needs on participation of the GP in this process (and how this should be arranged)	FG1/FG2

6. Potential benefits/pitfalls

Sample questions:

1. How do you feel about e-consultation? Are there benefits or pitfalls? FG1/FG2
2. How do you feel about treatment as part of clinical trials? FG1/FG2
3. Should e-consultation be offered for each patient with cancer (i.e. CRLM)? Or only in a sub-group of patients? And which patients may qualify for this subgroup? FG1

Information needed:

Potential benefits and/or pitfalls of e-consultation services	FG1/FG2
Views on treatment as part of clinical trials	FG1/FG2
Patient qualification for e-consultation	FG1
