# Supplementary figures and images for: Perspectives of patients with colorectal cancer liver metastases on e-consultation in transmural care: a qualitative study: Is privacy really an issue?
Source: BMC Health Serv Res. 2023 May 25;23:541. doi: 10.1186/s12913-023-09408-5 (PMC10209560; doi:10.1186/s12913-023-09408-5)

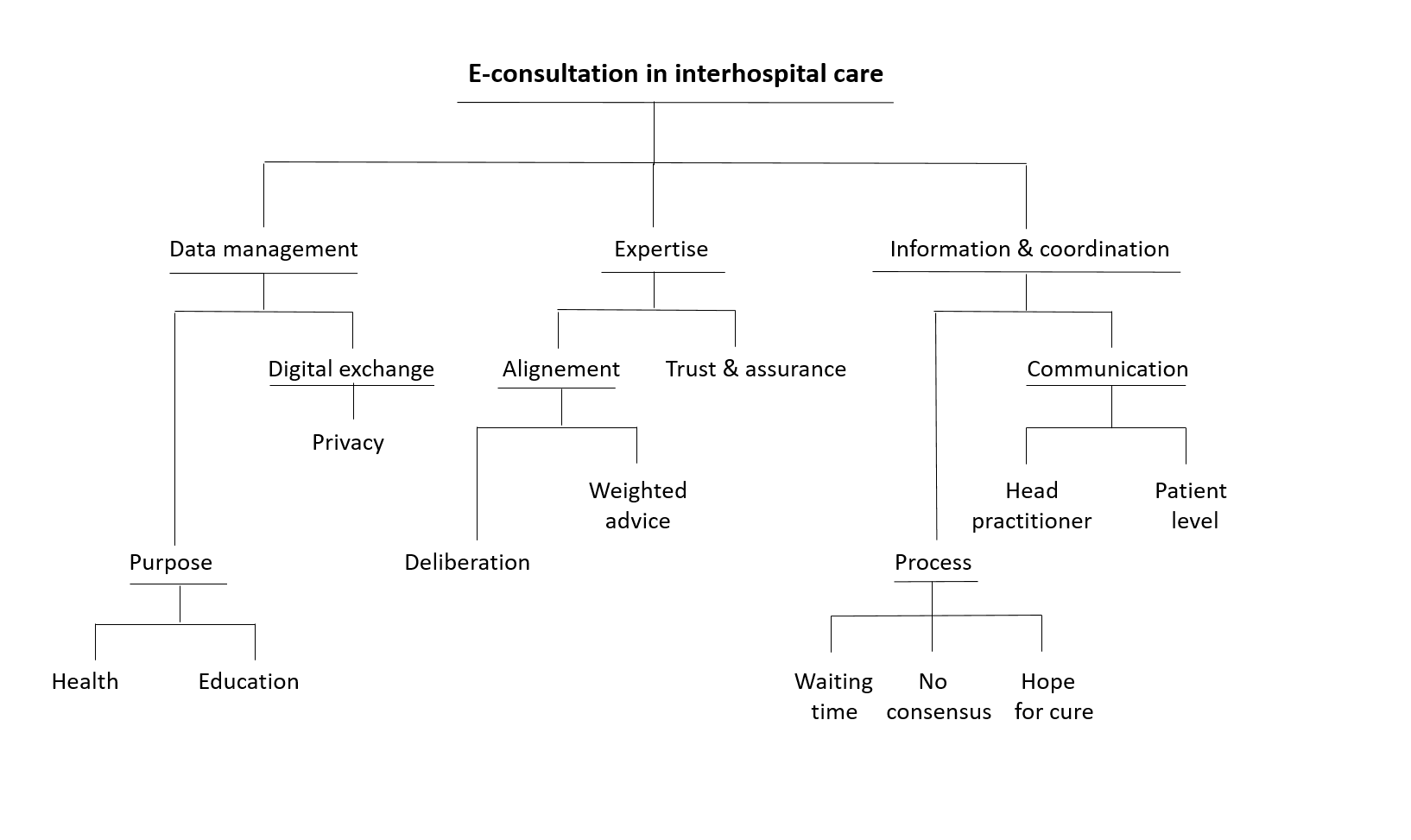

Supplement: Supplementary file 3 — Supplementary Material 3 [file 12913_2023_9408_MOESM3_ESM.tif]
